# Zero-gravity microbiome vortices: decoding orbital sepsis mimics and probiotic dysregulation in space health systems

**DOI:** 10.1097/MS9.0000000000004299

**Published:** 2025-11-11

**Authors:** Ureba Memon, Dua Memon, Muhammad Talha, Mahnoor Fatima

**Affiliations:** a Department of Medicine, Liaquat University of Medical and Health Sciences, Jamshoro, Pakistan; b Department of Medicine, King Edward Medical University, Lahore, Pakistan; c Department of Medicine, Shaheed Ziaur Rahman Medical College, Rajshahi Medical University, Bogura, Bangladesh

*To the Editor,*

Human microbiome stability is the basis of astronaut physiology; it directly affects the immune system, metabolism, and neurobehavioral health under extreme space conditions^[1]^. Microgravity and solar radiation significantly change microbial communities, which in turn increase virulence, resistance, and metabolite production that cause imbalance of the organism’s natural state^[2]^. The evidence of the International Space Station (ISS) missions reveals that the dysbiosis that occurs is similar to inflammatory disorders that happen on Earth; the decrease of *Lactobacillus* and *Bifidobacterium* impairs immune regulation^[1]^. Such changes lead to the formation of “microbiome vortices,” fluctuating microbial ecosystems that may result in immune system activation similar to orbital sepsis mimics^[2]^. To neutralize such weaknesses, the use of blockchain-tracked probiotic devices has been put forward for individualized microbial surveillance and to guarantee biosafety in the next very long-duration missions^[1]^. This is in line with the TITAN Guidelines on the need for transparency in AI use in health care^[3]^.

Microgravity significantly changes microbial gene expression, which leads to the increase of virulence factors and resistance determinants due to stress-induced regulatory changes that impact the host-microbe balance^[2]^. Research on the ISS showed that biofilm density was increased, quorum-sensing was changed, and plasmid exchange among microbial communities in orbit was elevated^[2]^. NASA’s metagenomic analyses unveiled that long-term space travel deeply impacts the microbiome of the astronauts, turning the beneficial commensals into pathogens^[4]^. Studies on low-shear modeled microgravity conditions revealed that microgravity could accelerate mutation rates and the activation of mobile genetic elements, thereby enabling microbes to survive on spacecraft surfaces^[2]^. These microecological changes may lead to immune dysregulation and inflammatory symptoms similar to those of systemic infection or “pseudo-sepsis,” thus making the diagnosis more difficult in space health care systems^[4]^.

In fact, sepsis-like symptoms plus inflammatory syndromes were among the diseases that astronauts have experienced, and no infectious pathogen was detected. The conditions have been explained as changes in host-microbe signaling due to disrupted gravity^[1]^. Besides, microgravity and radiation are said to be the main factors behind the so-called “microbiome vortices,” i.e., rapid changes in the ecological system that result in the increase in the expression of virulence genes and metabolic noise of the resident flora^[2]^. There is microbial turbulence that interferes with immune balance, causes cytokine storms, and systemic inflammation resembling orbital sepsis, thus explaining the absence of sepsis in sterile cultures^[4]^. Anyway, this pseudo-septic physiology poses obstacles to diagnostic algorithms, whereby the existence of pathogens is the basis for sepsis already detected while dysbiosis biomarkers are not considered^[1]^. Unraveling these orbital sepsis mimics is a matter of utmost importance for space medics to be able to tell the difference between a real infection and inflammation caused by the microbiome in deep-space missions to come^[2]^.

Probiotic tracking on the blockchain can be to microbe standard from pre-flight to post-flight by recording the provenance data of the strains like *Bacillus clausii* that were used in SAP-1 payload experiments^[5]^. Decentralized ledgers may record culture viability, radiation exposure, and nutrient conditions communicated from SAP-1’s onboard communication subsystems^[5]^. During solar flare events, changes in ionizing radiation that interfere with microbial metabolism and sensor telemetry cause “failure cascades” in growth analytics as these cascades propagate along the instrument chain^[5]^. By combining blockchain with these on-the-fly biomedical systems, the transparency of astronaut health datasets would be ensured, and the stability of probiotics against microgravity-induced dysbiosis would be confirmed, thus paving the way for secure, auditable monitoring frameworks for extraterrestrial life-support missions^[5,6]^.

Solar Particle Events represent temporary but very powerful sources of ionizing radiation. They can deliver extremely high dose rates, an example being the 1989 event estimated to reach 1454 mGy/h, that are superimposed on the chronic galactic cosmic ray background. This radiation does damage through direct ionization, which is the most effective way to break chemical bonds and cause DNA damage. The interaction also liberates a variety of free radical species, among them radical oxygen ions and hydrogen peroxide formed in water radiolysis, thus leading to very high oxidative stress. In probiotics, this may cause microbial genetic drifts resulting in the loss of probiotic viability and functionality. The radiation-induced damage to human cells and essential microbial flora accumulated over time can be one of the causes of systemic immune dysregulation that may appear as sepsis-mimic syndromes in astronauts^[7]^.

In space travel, infection prevention and antimicrobial stewardship are riddled with challenges that are brought about by changes in the immune system of the body, increased bacterial virulence, and reduced diagnostic capabilities in the modules of the spacecraft. Dysbiosis and changes in the astronaut microbiome, including decreases in beneficial *Lactobacillus* and *Bifidobacterium* and increases in opportunistic pathogens, make differential diagnosis of infection-like symptoms more difficult^[6]^. The limitation of lab diagnostics makes the use of predictive methods more prominent and also shows the potential of AI-assisted biosurveillance in interpreting the changing microbiome data for quick intervention^[2]^. The use of probiotics can be a source of energy for the immune system and also help the microbial homeostasis, thus infection prevention can be achieved in space^[2,6]^.

Enhancing health systems for astronauts in deep space missions necessitates bridging technology and research gaps that are critical. There have been no studies on the viability and effectiveness of probiotics that have been stored in space for a long time, thereby making it a major challenge to figure out how to stabilize them in a microgravity environment. Deciphering the changes in gut microbiota composition due to spaceflight, together with the increase in the pathogenicity of microorganisms and antibiotic resistance, calls for advanced genomic analysis and the implementation of real-time monitoring systems. The coming era of space probiotics will depend on radiation-resistant probiotic development once it is understood that certain probiotics, like *B. clausii*, have a natural resistance to space radiation. It is fundamental to set up extensive reference data from both the earth and space microbiomes so that the microbial adaptation and environmental impacts can be entirely clarified. The current studies suggest that probiotics can play a key role in the alleviation of space-induced stress as well as other gastrointestinal symptoms; thus, they should continuously be investigated in terms of their behavior and viability^[5]^.

Research on the space microbiome is leading to critical findings that have a major impact on the Earth’s health care system and are driving global health care innovation. Changes in the gut microbiota of astronauts are one of the direct examples of such findings, where such a change is the increase of *Parasutterella* sp, a bacterium associated with inflammatory bowel disease; these changes help the doctors on Earth to decide the correct treatments for gut diseases^[6]^. Knowing the increased pathogenic potential and antibiotic resistance that occur in space can be a great weapon in the fight against the causes of harsh infections on Earth and also prevent infections that arise during intensive care, for example, the so-called “ICU sepsis mimics”^[5]^. Probiotics, which are necessary for the regulation of the gut of an astronaut and proper functioning of the immune system^[6]^, can work as a prototype for microbiome-based treatments on Earth^[5]^. The space discoveries are opening the way to a greater understanding of the physiological changes and are eventually helping to progress biomedicine on Earth^[6]^.

The disruption of the microbiota during the space mission, which is characterized by changed microbial populations and increased pathogenic potential, is cited by the authors as a source of risks similar to orbital sepsis mimics^[1,5,6]^. These intricate health problems of the space crew can be resolved with the aid of complete strategies, such as continuous microbial monitoring and sophisticated pathogen control systems, to guarantee the crew’s safety^[1]^. There is no clear mention of “digital probiotic tracking” as a specific entity in the articles that have been given, but the need for strong systems to oversee microbial interventions and health is obvious. We put forward the integrated systems and the multidisciplinary approach, which draws the combination of astrobiology, infectious disease, and data science to ensure not only microbial but also crew resilience, thus, astronaut health is a critical factor that is safeguarded for future long-duration space missions^[1,5,6]^.

## Data Availability

Not applicable to this study.
